# Multi-omics analysis reveals the effects of host-rumen microbiota interactions on growth performance in a goat model

**DOI:** 10.3389/fmicb.2024.1445223

**Published:** 2024-09-09

**Authors:** Juncai Chen, Xiaoli Zhang, Xuan Chang, Bingni Wei, Yan Fang, Shanshan Song, Daxiang Gong, Deli Huang, Yawang Sun, Xianwen Dong, Yongju Zhao, Zhongquan Zhao

**Affiliations:** ^1^College of Animal Science and Technology, Chongqing Key Laboratory of Herbivore Science, Southwest University, Chongqing, China; ^2^Tengda Animal Husbandry Co., Ltd., Chongqing, China; ^3^Chongqing Academy of Animal Science, Chongqing, China

**Keywords:** microbes, rumen epithelium, transcriptome, growth rate, goat

## Abstract

The growth rate of young ruminants has been associated with production performance in later life, with recent studies highlighting the importance of rumen microbes in supporting the health and growth of ruminants. However, the specific role of rumen epithelium bacteria and microbiota-host interactions in influencing the early life growth rate of ruminants remains poorly understood. In this study, we investigated the rumen fermentation pattern, microbiota characteristics, and global gene expression profiles of the rumen epithelium in 6-month-old goats with varying growth rates. Our results showed that goats with high average daily gain (HADG) exhibited higher rumen propionate concentrations. Goats with low average daily gain (LADG) had the higher relative abundances of rumen epithelium bacteria genera U29-B03 and *Quinella*, while exhibiting a lower relative abundance of *Lachnospiraceae* UCG-009. In the rumen fluid, the relative abundances of bacteria genus *Alloprevotella* were lower and *Desulfovibrio* were higher in LADG goats compared to HADG goats. Additionally, the relative abundance of fungal genus *Symmetrospora* was lower in LADG goats compared to HADG goats. Transcriptome analysis showed that 415 genes were differentially expressed between LADG and HADG goats, which were enriched in functions related to cell junction and cell adhesion, etc. Correlation analysis revealed that rumen epithelium bacteria genera *UCG-005* and Candidatus *Saccharimonas* were negatively associated, while *Lachnospiraceae* NK3A20 group and *Oscillospiraceae* NK4A214 group were positively associated with average daily gain (ADG) and genes related to barrier function. The rumen fluid bacteria genus *Alloprevotella* was positively correlated, while *Desulfovibrio* was negatively correlated with rumen propionate and ammoniacal nitrogen (NH_3_-N) concentrations, as well as genes related to barrier function and short chain fatty acids (SCFAs) transport. In summary, our study reveals that the higher ruminal fermentation efficiency, improved rumen epithelial barrier functions, and enhanced SCFAs transport in HADG goats could be attributed to the rumen microbiota, particularly the rumen epithelium bacteria, such as *Lachnospiraceae* and *Oscillospiraceae* NK4A214 group.

## Introduction

The rumen constitutes a complex natural ecosystem that harbors diverse microbes responsible for biomass degradation, which provide nutrients for the host’s physiological needs. In return, the rumen provides a unique environment characterized by anaerobic conditions, stable temperature, and high osmotic pressure for the rumen microbes (Kamra, 2005). Emerging evidence suggests that the stable mutualistic relationship between rumen and its microbial inhabitants is pivotal for the growth, development (Malmuthuge et al., 2019; Jize et al., 2022), feed efficiency (Fonseca et al., 2023), and mitigation of acidosis (Li et al., 2019) in ruminants. However, these studies predominantly focused on rumen fluid-associated bacteria, which are more sensitive to the changes in environmental conditions (Liu et al., 2016) and differ from rumen epithelium bacteria (Pei et al., 2010). Rumen epithelium bacteria, directly adhering to the epithelium, likely engage in more interactions with the host. Moreover, fungi are immensely important for degrading ligno-carbohydrate complex in rumen (Bhagat et al., 2023). Yin et al. (2023) identified several fungal biomarkers in both rumen and rectum that were associated with growth rate in lambs. However, our understanding of the functional roles of rumen fungi remains limited and further investigations are required.

Early-life growth rate significantly influences later-life production performance. The pre-weaning and pre-pubertal average daily gain (ADG) has been reported to be correlated with milk production (Soberon and Van Amburgh, 2013; Gelsinger et al., 2016) and fertility (Schubach et al., 2019; Costa et al., 2021) in ruminants. There is a growing interest in exploring key microbes that affect the health and growth performance of ruminant animals. Yin et al. (2023) identified several microbes in both the rumen and the rectum associated with post-weaning ADG in lambs. The ADG-related rumen fluid microbiota, including the *Prevotellaceae* family, *Streptococcus*, and Candidatus *Saccharimonans*, is believed to contribute to nutrient metabolism functions and short chain fatty acids (SCFAs) production in dairy goats (Wang et al., 2023). Nevertheless, the role of rumen epithelium bacteria and microbiota-host interactions in influencing the early-life growth performance of goats remains unclear.

The objective of the present study was to investigate the relationships between ruminal microbiota and early-life growth performance in ruminants, and to further explore the underlying mechanisms involved in the microbiota-host interactions at transcriptome level. Here, 6-month-old goats with different ADG, raised under identical conditions, were selected as model animals to assess their rumen fermentation patterns, rumen microbiota characteristics, and global gene expression profiles of the rumen epithelium. The findings of this study may contribute to the development of novel strategies aimed at improving the growth rate of young ruminants, thereby potentially enhancing production performance in later life.

## Materials and methods

### Ethics statement

All animal experimental and protocols were approved by the Institutional Animal Care and Use Committee of Southwest University, Beibei, China (approval IACUC-20220630-04).

### Animal experiment design

A total of 49 female Dazu black kids from Tengda Animal Husbandry Co., Ltd. (Chongqing, China) were used for the experiment. Birth weights of all goat kids were recorded immediately after birth and they were weaned at 2 months of age. The goats were fed with a total mixed ration (TMR) *ad libitum* twice daily at 0700 and 1,600, consisting of corn grain (475 g/kg), soybean meal (40 g/kg), alfalfa hay (450 g/kg), NaCl (5 g/kg), dicalcium phosphate (10 g/kg), sodium bicarbonate (10 g/kg), and a trace minerals and vitamins supplement (10 g/kg). The diet was antibiotic-free, meeting the recommendations of the Feeding Standard of Meat-producing Sheep and Goats of China, NY/T 816–2004 (Ministry of Agriculture, MOA, PRC, 2004).

At approximately 6 months of age (183.4 ± 6.2 days), the goats were weighed before morning feeding and then slaughtered after anesthesia. The ADG was calculated as the weight gain from birth to 6 months of age divided by the number of days. The average ADG for all 49 goats was 86.8 ± 10.9 g/day (Supplementary Table S1), after which goats were ranked based on individual ADG. The top 10% of goats were designated as the high ADG group (HADG, 99.8 ± 12.8 g/day), while the bottom 10% were designated as the low ADG group (LADG, 63.6 ± 3.94 g/day).

Dry matter intake was continuously measured for 2 days before slaughtering (844 ± 28 g/day for LADG goats and 857 ± 37 g/day for HADG goats, *p* = 0.55). The rumen fluid was filtered using a four-layer cheesecloth, and pH values were measured with a portable pH meter (PHB-4, Rex Instrument, Shanghai, China). Rumen epithelium samples were collected from the ventral sac of each goat immediately after slaughter and rinsed with 0.01 M phosphate-buffered saline (PBS) buffer. All samples were promptly immersed in liquid nitrogen and then stored at −80°C for subsequent analysis.

### The SCFAs and NH_3_-N analysis

The rumen fluid was centrifuged for 10 min at 10,000 × g, and the supernatants were filtered with a membrane (0.22 μm). Acetate, propionate, and butyrate concentrations were determined using high-performance liquid chromatography (KC-811 column, Shodex; mobile phase, 3 mM perchloric acid; flow rate, 1.0 mL/min; temperature: 50°C). Ammonia-N (NH_3_-N) concentration was determined using the phenol-hypochlorite colorimetric method.

### Rumen microbiota analysis

Total microbial DNA was extracted from rumen fluid and epithelial samples using the E.Z.N.A.^®^ stool DNA Kit (Omega Bio-tek, Norcross, GA, U.S.) according to the manufacturer’s protocol. The quality of the DNA was determined by 1% agarose gel electrophoresis. Bacterial 16S rRNA gene fragments (V3-V4) were amplified with primers 338F (5’-ACTCCTACGGGAGGCAGCAG-3′) and 806R (5’-GGACTACHVGGGTWTCTAAT-3′), and fungi ITS rRNA gene was amplified with primers ITS1F (5’-CTTGGTCATTTAGAGGAAGTAA-3′) and ITS2R (5’-GCTGCGTTCTTCATCGATGC-3′) using an ABI GeneAmp^®^ 9,700 PCR thermocycler (ABI, CA, United States). The amplicons were paired-end sequenced using the Illumina PE250 platform (Illumina, San Diego, USA). The raw sequencing reads were deposited in the NCBI Sequence Read Archive (SRA) database (Accession Number: PRJNA1099693). The resulting sequences were filtered using FASTP (version 0.19.6) and merged using FLASH (version 1.2.11). The amplicon sequence variant (ASV), obtained using DADA2 in QIIME2 (version 2020.2), were assigned to taxonomies using SILVA database (version 138) and UNITE database (version 8.0) for bacteria and fungi, respectively. The alpha diversity indices, including Chao1, ACE, Sobs, Shannon, and Simpson, were analyzed using Mothur (version 1.30.2). Principal coordinate analysis (PCoA) was performed based on weighted UniFrac distances and significance was determined using ANOSIM with 999 permutations.

### Transcriptome analysis

Total RNA was extracted from rumen epithelium using Trizol reagent (Tiangen Biotech, Beijing, China) according to the manufacturer’s protocol. RNA quality was determined by a 5,300 (Bioanalyser Agilent, Palo Alto, CA, United States) and high-quality RNA samples were sent to Majorbio Biotech (Shanghai, China) for commercial library preparation and sequencing on the Illumina Novaseq 6,000. The DEGs (differential expression genes) between groups were analyzed using DESeq2. Genes with |log_2_FC| ≥ 1 and FDR < 0.05 were considered as differentially expressed. The gene ontology (GO) functional-enrichment analyses were performed by Goatools to identify significantly enriched GO terms at a Bonferroni-corrected *p*-value ≤0.05. RNA sequencing data were deposited in the NCBI’s Gene Expression Omnibus under the accession number PRJNA1101717.

### Tissue RNA extraction and real-time quantitative PCR

Total RNA was extracted from rumen epithelium using Trizol reagent (Tiangen Biotech, Beijing, China) according to the manufacturer’s protocol. RNA degradation and contamination were monitored through 1% agarose gels electrophoresis. RNA concentration and purity were examined using a NanoDrop spectrophotometer (NanoDrop Technologies, Wilmington, DE, United States). The gene expression was detected by quantitative real-time PCR (RT-qPCR) using the 2^−∆∆CT^ method. The primer sequences are listed in Supplementary Table S2. β-actin and GAPDH were used as the endogenous reference genes. All reactions were run in triplicate for each sample.

### Statistical analysis

Statistical significance was assessed using an unpaired Student’s two-tailed t-test for average daily gain, ruminal pH, NH_3_-N, and SCFAs, or the one-way ANOVA with Tukey’s *post hoc* tests for alpha diversity of the bacterial community, using GraphPad Prism (version 9.2.0, GraphPad Software, San Diego, CA, United States). Results are expressed as means with the standard error of the means (SEM). A *p*-value of less than 0.05 was considered statistically significant. Correlations between rumen microbiota and growth performance, ruminal SCFAs and NH_3_-N, or gene expression were estimated by Spearman correlation analysis using the ‘Hmisc’ and ‘Corrplot’ packages in R (version 4.3.2).

## Results

### Ruminal pH, NH_3_-N, and short-chain fatty acids concentrations

**Figure 1:**
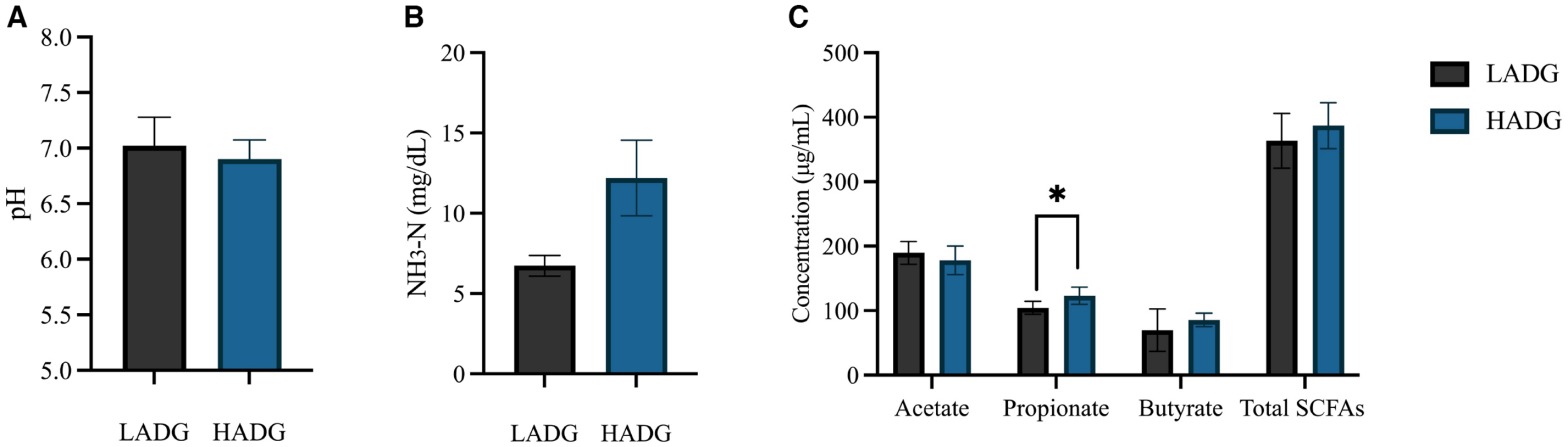
The ruminal pH **(A)**, NH3-N **(B)**, and short-chain fatty acids (SCFAs) concentrations **(C)** of low average daily gain (LADG) or high average daily gain (HADG) goats. * *p* < 0.05.

HADG goats had higher rumen propionate concentrations (*p* < 0.05) and tended to have higher rumen NH_3_-N concentration (*p* = 0.07, Figure 1). However, no significant differences were found in rumen fluid pH, and ruminal acetate and butyrate concentrations between HADG and LADG goats.

### Microbiota composition of rumen fluid and epithelium

In the current study, 16 s and ITS gene sequencing techniques were used to determine the differences in rumen microbiota, especially for rumen epithelium bacteria, between HADG and LADG goats, aiming to explore the potential microbial targets for the development of novel intervention strategies to improve the growth performance of young ruminants.

The alpha diversity of the bacterial community in rumen fluid and epithelium is shown in Table 1. Bacterial community richness indices (Chao1, Ace, and Sobs) were greater in rumen epithelium of both HADG and LADG goats compared with rumen fluid of the same groups (*p* < 0.01). Nonetheless, no significant difference was observed in bacterial community composition between HADG and LADG goats in either rumen fluid or epithelium. In rumen fluid, the bacterial diversity of HADG was higher than that of LADG (*p* < 0.01), as indicated by Shannon indices.

**Table 1:** The alpha diversity of rumen fluid or rumen epithelium microbiota in goats.

Items	RE-LADG	RE-HADG	RF-LADG	RF-HADG	*p* values
ACE index	953.6 ± 347.0 ^a^	931.8 ± 265.3 ^a^	225.4 ± 31.3 ^b^	224.7 ± 42.4 ^b^	< 0.01
Chao1 index	898.7 ± 353.5 ^a^	866.4 ± 196.1 ^a^	225.4 ± 31.3 ^b^	225.0 ± 42.7 ^b^	< 0.01
Sobs index	624.6 ± 184.6 ^a^	635.6 ± 104.4^a^	224.0 ± 30.5 ^b^	223.2 ± 42.1 ^b^	< 0.01
Shannon index	4.88 ± 0.33 ^a^	5.05 ± 0.28 ^a^	4.07 ± 0.58 ^b^	4.60 ± 0.34 ^a^	< 0.01
Simpson index	0.022 ± 0.003 ^b^	0.018 ± 0.006 ^b^	0.085 ± 0.085 ^a^	0.027 ± 0.024 ^ab^	0.04

Significant differences were tested by one-way ANOVA with Tukey’s post hoc tests (*n* = 5 per group). The variant letter indicates a significant difference when *P* < 0.05. RE-LADG, rumen epithelium of low average daily gain goat; RE-HADG, rumen epithelium of high average daily gain goat; RF-LADG, rumen fluid of low average daily gain goat; RF-HADG, rumen fluid of high average daily gain goat.

**Figure 2:**
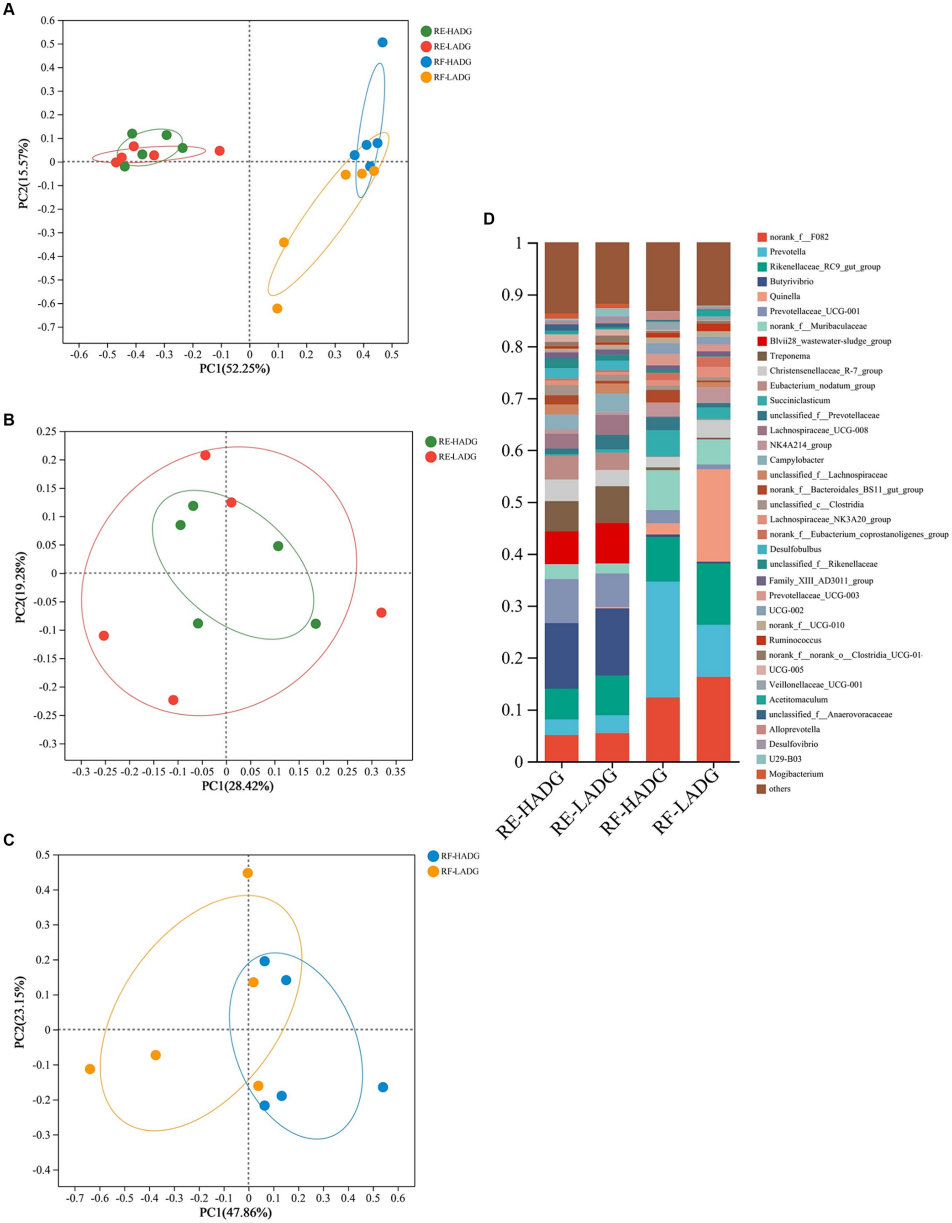
Principal coordinate analysis (PCoA) of bacterial compositional profiles of all samples **(A)**, rumen epithelium samples **(B)**, and rumen fluid samples **(C)** between LADG and HADG goats, based on weighted UniFrac distances. Abundant genera **(D)** in the rumen bacteria of LADG or HADG goats. Data are shown as means. RE-LADG, rumen epithelium of low average daily gain goat; RE-HADG, rumen epithelium of high average daily gain goat; RF-LADG, rumen fluid of low average daily gain goat; RF-HADG, rumen fluid of high average daily gain goat.

As shown in Figure 2A, bacterial communities of rumen epithelium were well separated from those in the rumen fluid, with 52.25 and 15.57% of the variations explained by principal component 1 (PC1) and PC2, respectively. The rumen fluid bacterial composition of LADG goats was separated from the HADG goats (Figure 2C). However, the rumen epithelium bacterial composition was similar between LADG and HADG goats (Figure 2B).

We found that the *Butyrivibrio* (12.6% in HADG goats and 12.9% in LADG goats), *Prevotellaceae* UCG-001 (8.5% in HADG goats and 6.5% in LADG goats), *Blvii28* wastewater-sludge group (6.3% in HADG goats and 7.3% in LADG goats), *Rikenellaceae* RC9 gut group (5.9% in HADG goats and 7.6% in LADG goats), *Treponema* (5.9% in HADG goats and 7.1% in LADG goats), and norank_f_F082 (5.0% in HADG goats and 5.4% in LADG goats) were predominant genera in the rumen epithelium (Figure 2D). In contrast, the *Prevotella* (12.6% in HADG goats and 12.9% in LADG goats), norank_f_F082 (12.3% in HADG goats and 16.2% in LADG goats), *Rikenellaceae* RC9 gut group (8.6% in HADG goats and 11.8% in LADG goats), *Quinella* (2.1% in HADG goats and 17.9% in LADG goats), norank_f_*Muribaculaceae* (7.8% in HADG goats and 4.8% in LADG goats), *Succiniclasticum* (5.1% in HADG goats and 2.2% in LADG goats) were predominant genus in the rumen fluid.

**Figure 3:**
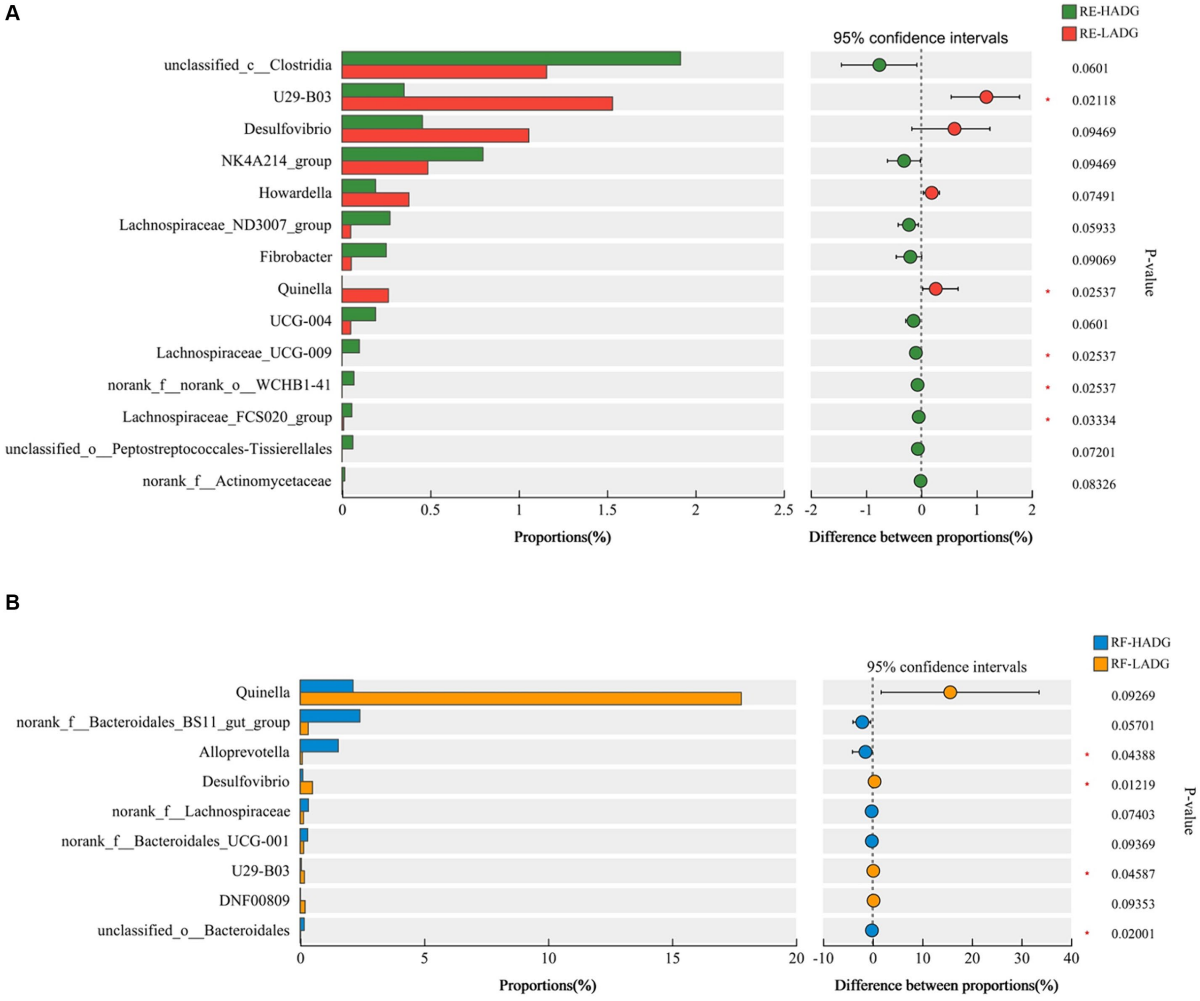
Differential enrichment of bacterial genera in rumen epithelium samples **(A)** and rumen fluid samples **(B)** between LADG and HADG goats, based on Wilcoxon rank-sum test. Data are shown as means. RE-LADG, rumen epithelium of low average daily gain goat; RE-HADG, rumen epithelium of high average daily gain goat; RF-LADG, rumen fluid of low average daily gain goat; RF-HADG, rumen fluid of high average daily gain goat.

In rumen epithelium, the relative abundances of genera U29-B03 and *Quinella* were higher, while *Lachnospiraceae*_UCG-009, norank_f_norank_o_WCHB1-41, and *Lachnospiraceae*_FCS020_group were lower in LADG goats compared with HADG goats (*p* < 0.05, Figure 3A). Additionally, the relative abundance of unclassified_c_*Clostridia* tended to be lower (*p =* 0.06), and *Desulfovibrio* tended to be higher (*p =* 0.09) in rumen epithelium of LADG goats compared with HADG goats. In rumen fluid, the relative abundances of genera *Alloprevotella* and unclassified_o_*Bacteroidales* were lower, and *Desulfovibrio* and U23-B03 were higher in LADG goats compared with HADG goats (*p* < 0.05, Figure 3B). Moreover, the relative abundance of norank_f_*Bacteroidales*_BS11_gut_group tended to be lower (*p =* 0.06), and *Quinella* (*p =* 0.09) tended to be higher in LADG goats compared with HADG goats.

**Figure 4:**
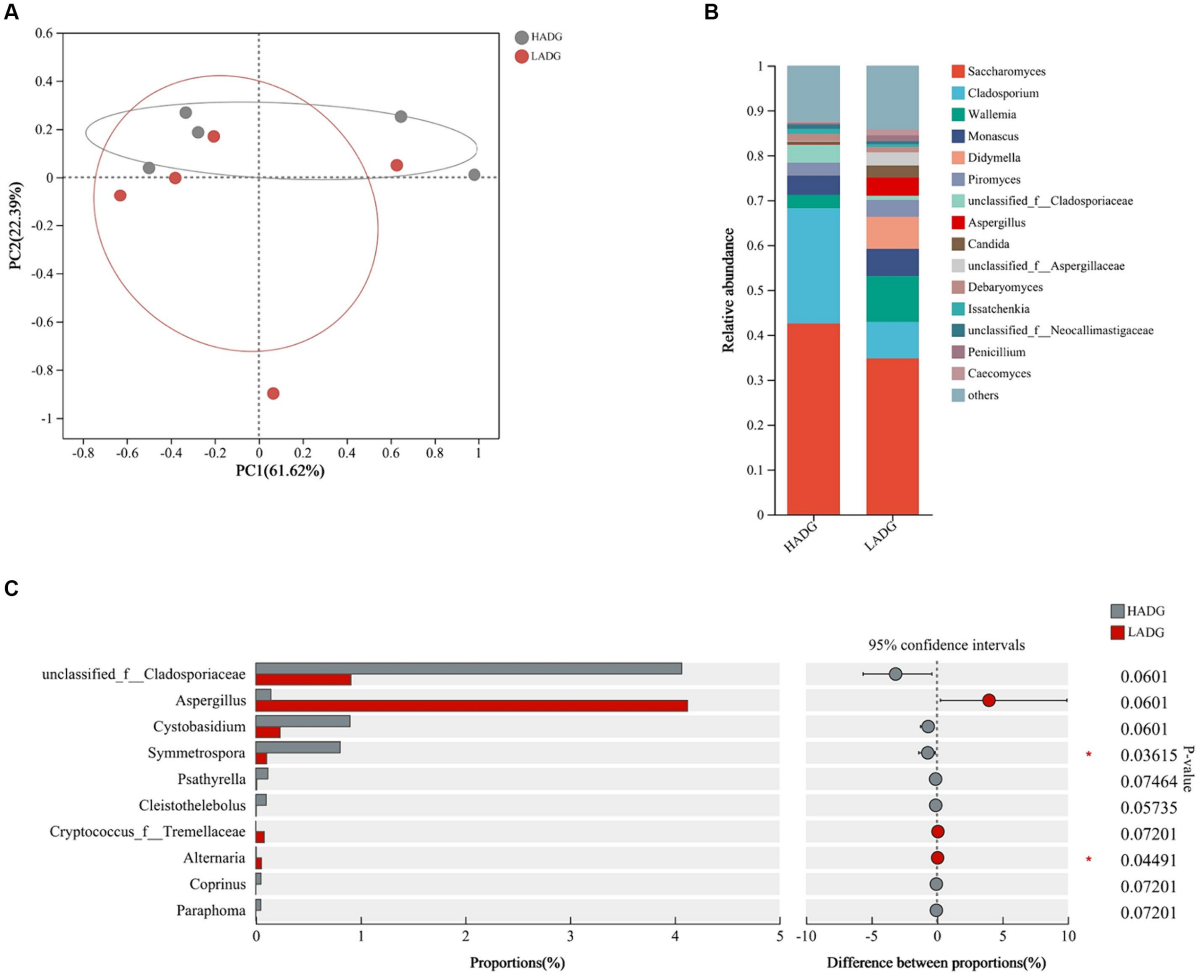
Principal coordinate analysis (PCoA) of fungal compositional profiles of rumen fluid samples between LADG and HADG goats, based on weighted UniFrac distances **(A)**. Abundant genera in the rumen fungi of LADG or HADG goats **(B)**. Differential enrichment of fungus genera of rumen epithelium samples **(C)** between LADG and HADG goats, based on Wilcoxon rank-sum test. Data are shown as means. *n* = 5 per group. LADG, low average daily gain goats; HADG, high average daily gain goats.

To explore whether rumen fungi were related to the growth performance, we analyzed the fungal compositions of rumen fluid in LADG and HADG goats (Figure 4). The *Saccharomyces* (42.6% in HADG goats and 34.8% in LADG goats), *Cladosporium* (25.7% in HADG goats and 8.1% in LADG goats), *Wallemia* (3.0% in HADG goats and 10.3% in LADG goats), and *Monascus* (4.4% in HADG goats and 6.0% in LADG goats) were predominant genera in the rumen fluid. Alpha diversity analysis showed that the rumen fluid fungal composition of LADG goats was not clearly separated from the HADG goats. However, the relative abundance of genus *Symmetrospora* was lower in LADG goats compared with HADG goats (*p* < 0.05). Additionally, the relative abundance of genus unclassified_f_*Cladosporiaceae* tended to be lower (*p =* 0.06), and *Aspergillus* (*p =* 0.06) tended to be higher in LADG goats compared with HADG goats.

### RNA-Seq analysis of the rumen epithelium

**Figure 5:**
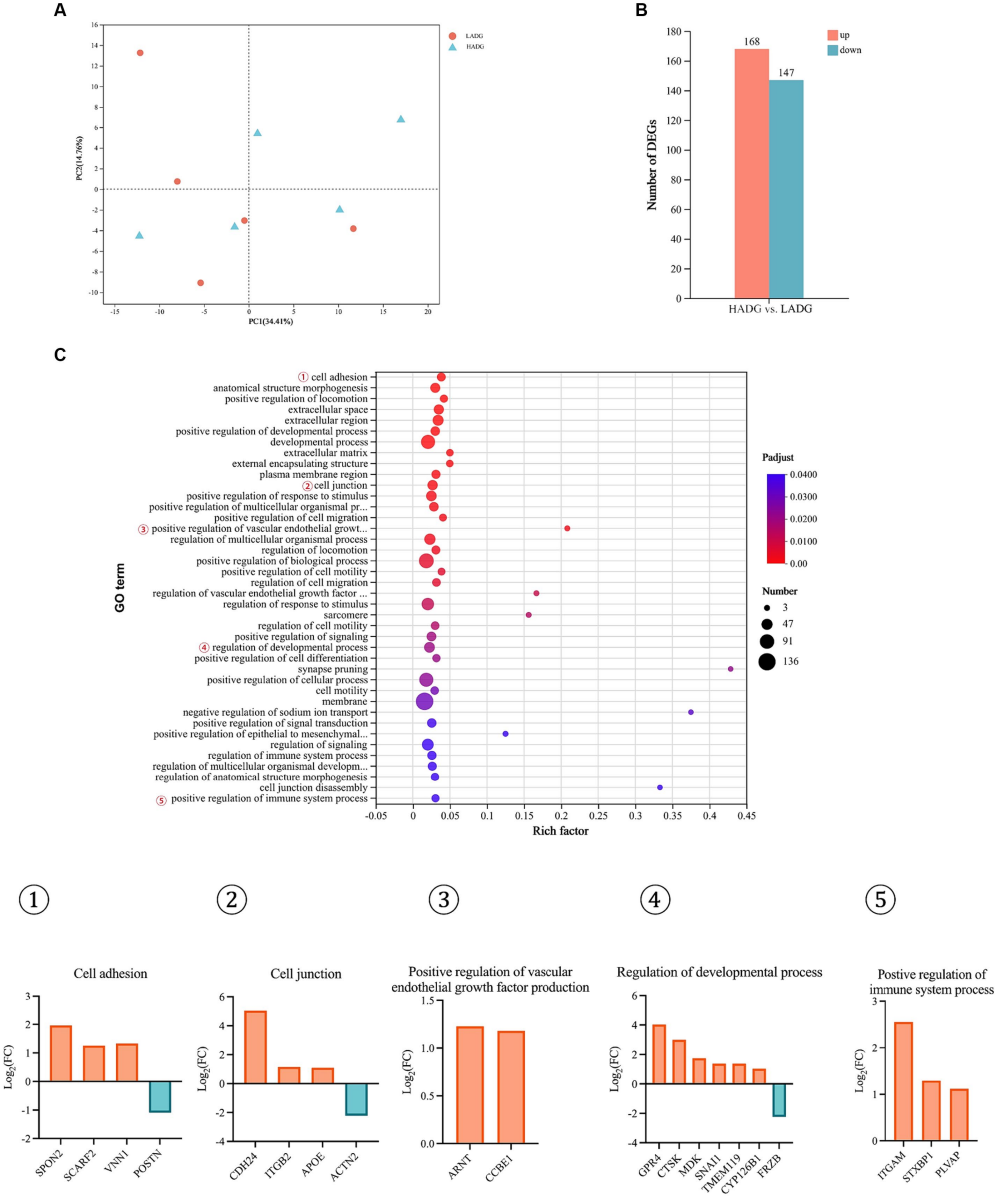
RNA-seq analysis of the rumen epithelium of HADG and LADG goats. Principal component analysis (PCA) of gene expressions in rumen epithelium between LADG and HADG goats **(A)**. The number of differentially expressed genes (DEGs) between HADG and LADG goats **(B)**. The GO pathways significantly enriched in the DEGs in rumen epithelium between LADG and HADG goats **(C)**. *n* = 5 per group. LADG, low average daily gain goats; HADG, high average daily gain goats.

Principal components analysis (PCA) did not reveal strong clustering, with 34.41 and 14.76% variations explained by PC1 and PC2, respectively (Figure 5A). Further analysis of differentially expressed genes (DEGs) showed that 168 DEGs were upregulated, while 147 DEGs were downregulated in HADG goats compared with LADG goats (Figure 5B). 65 GO terms, including cell adhesion, cell junction, positive regulation of vascular endothelial growth factor production, regulation of developmental process, positive regulation of immune system process, etc., were identified (Figure 5C). Specifically, three DEGS, *SPON2*, *SCARF2*, *VNN1*, were upregulated and *POSTN* was downregulated, which were involved in regulation of cell adhesion, in HADG goats compared with LADG goats. Three upregulated genes (*CDH24*, *ITGB2, APOE*) and one downregulated gene (*ACTN2*) were enriched in the GO terms of cell junction in HADG goats. The *ARNT* and *CCBE1* involved in regulation of vascular endothelial growth factor production were upregulated in HADG goats. Five upregulated genes (*GPR4*, *CTSK*, *MDK*, *SNAI1*, *TMEM119*, *CYP126B1*) and 1 downregulated gene (*FRZB*) were enriched in the GO terms of regulation of developmental process in HADG goats. Additionally, three genes (*ITGAM*, *STXBP1*, and *PLVAP*) involved in regulation of immune system process were upregulated in HADG goats.

### Relative gene expression of the rumen epithelium

**Figure 6:**
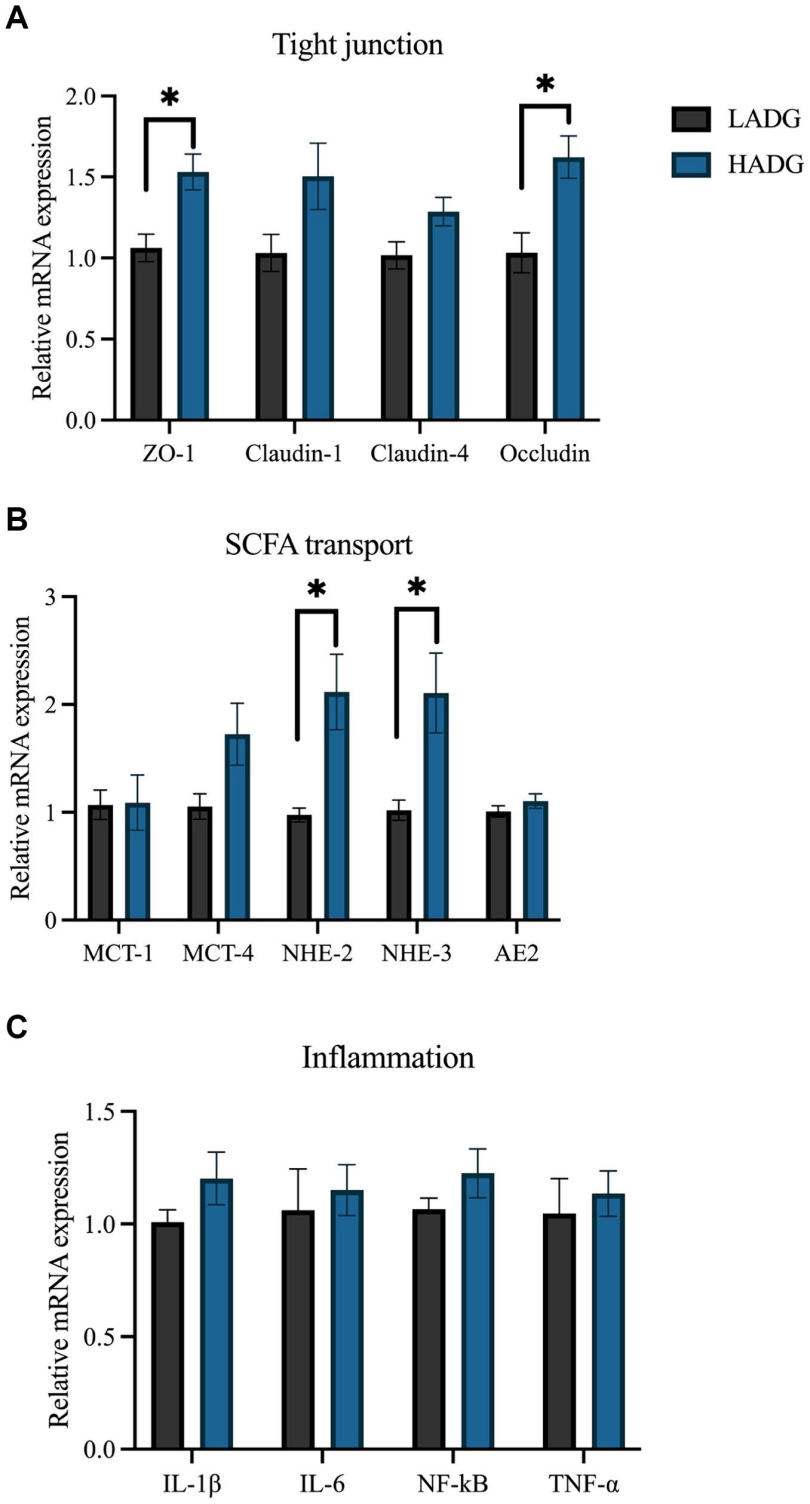
RT-PCR analyses of the relative mRNA expression of genes associated with tight junction **(A)**, short-chain fatty acids (SCFAs) transport **(B)**, and inflammation **(C)**. The values represent the mean ± SD (*n* = 5 per group). *p < 0.05.

The relative mRNA expression levels associated with tight junction, SCFAs transport, and inflammation are shown in Figure 6. The results demostrated that the relative expressions of *ZO-1* (*p* = 0.01), *Occludin* (*p* = 0.02), *NHE-2* (*p* = 0.03), and *NHE-3* (*p* = 0.03) were higher in the rumen epithelium of HADG goats compared with LADG goats. Furthermore, the relative expressions of *Claudin-1* (*p* = 0.10), *Claudin-4* (*p* = 0.09), and *MCT-4* (*p* = 0.08) tended to increase in the rumen epithelium of HADG goats compared with LADG goats. However, the relative mRNA expressions related to inflammation did not show significant differences between the two groups.

### Correlation analyses of the rumen microbiota, growth performance, rumen SCFAs and NH3-N concentrations, or gene expression levels

**Figure 7:**
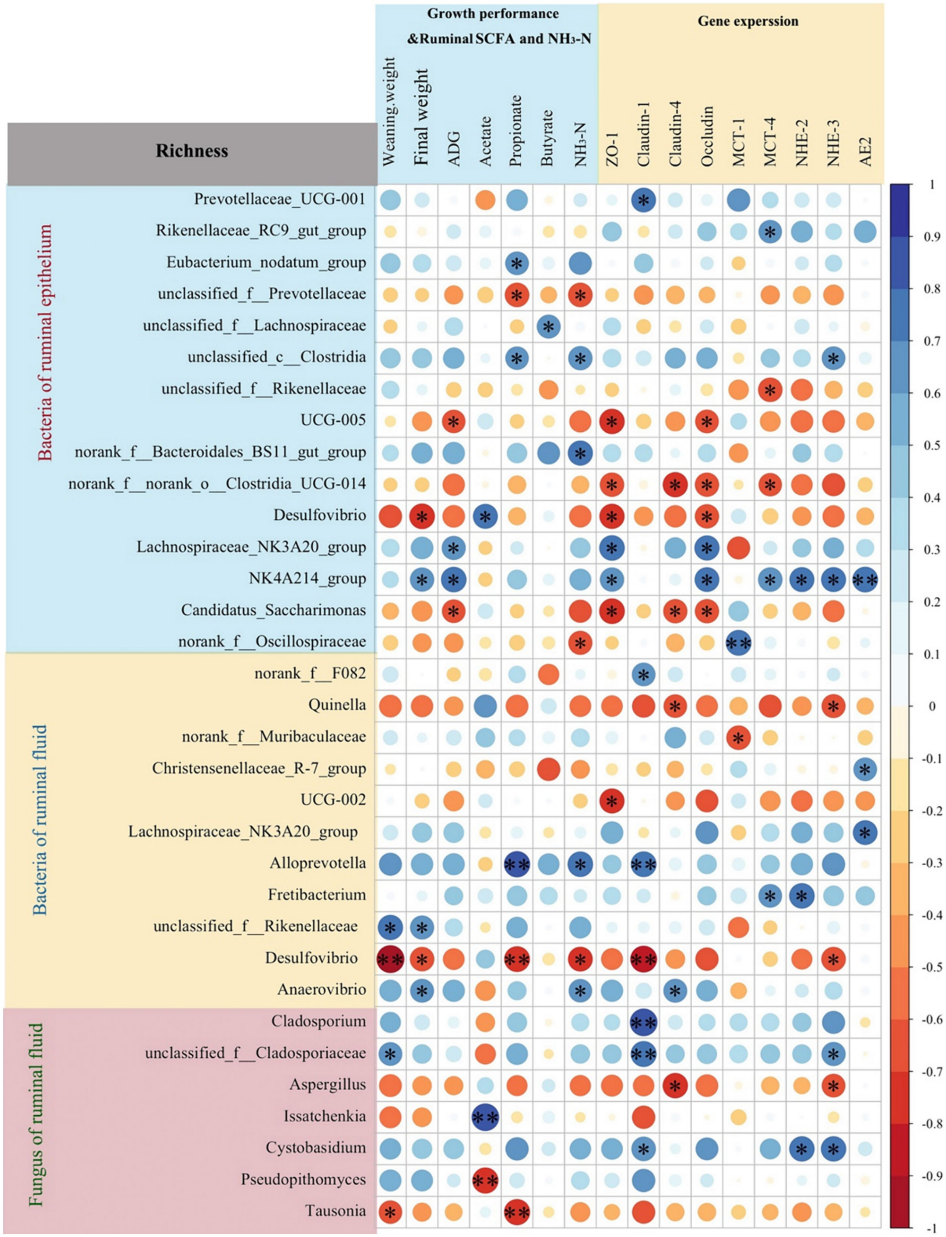
Spearman correlation analysis between rumen microbiota richness (fungi and bacteria of rumen fluid, and bacteria of rumen epithelium) and growth performance, ruminal short-chain fatty acids (SCFAs) and NH3-N concentrations, or gene expression levels. *p < 0.05, **p < 0.01.

The Spearman correlation analysis revealed that the relative abundances of genera *UCG-005* and Candidatus *Saccharimonas* in rumen epithelium were negatively associated with ADG, and relative expressions of *ZO-1* and *Occludin* (*p* < 0.05, Figure 7). The relative abundances of genera *Lachnospiraceae* NK3A20 group and *Oscillospiraceae* NK4A214 group in the rumen epithelium were positively correlated with ADG and the relative expressions of *ZO-1* and *Occludin* (*p* < 0.05). Additionally, the relative abundances of genera norank_f_norank_o_*Clostridia*_UCG-014 and *Desulfovibrio* in rumen epithelium were negatively associated with the relative expressions of *ZO-1* and *Occludin* (*p* < 0.05), while the relative abundance of genus *Oscillospiraceae* NK4A214 group in the rumen epithelium was positively associated with the relative expressions of *MCT-4*, *NHE-2*, *NHE-3*, and *AE2* (*p* < 0.05).

In rumen fluid, the relative abundance of *Quinella* had a negative correlation with the relative expressions of *claudin-4* and *NHE-3* (*p* < 0.05). Notably, the relative abundances of *Alloprevotella* in rumen fluid had a positive correlation with rumen propionate (*p* < 0.01) and NH_3_-N (*p* < 0.05) concentrations, and the relative expressions of *Claudin-1* (*p* < 0.01). Conversely, the relative abundance of *Desulfovibrio* in rumen fluid had a negative correlation with weaning weight (*p* < 0.01), rumen propionate (*p* < 0.01) and NH_3_-N (*p* < 0.05) concentrations, and the relative expressions of *ZO-1* (*p* < 0.01) and *NHE-3* (*p* < 0.05).

Furthermore, the relative abundances of fungal genera *Cladosporium* (*p* < 0.01), unclassified_f_*Cladosporiaceae* (*p* < 0.01), and *Cystobasidium* (*p* < 0.05) showed positive correlations with the relative expressions of *Claudin-1*. The relative of fungal genus *Issatchenkia* were positively (*p* < 0.01), and *Pseudopithomyces* were negatively (*p* < 0.01) associated with rumen acetate concentration.

## Discussion

We observed distinct rumen fermentation patterns between LADG and HADG goats, as reflected by lower rumen propionate and NH_3_-N concentrations in LADG goats. Propionate, a crucial precursor for gluconeogenesis in ruminants, is known to consume hydrogen and contribute to higher energy utilization efficiency and reduced methane formation in the rumen (Moss et al., 2000; Goopy et al., 2011). Shabat et al. (2016) further demonstrated that efficient ruminants exhibit higher ruminal propionate concentrations. Moreover, the tendency of higher rumen NH_3_-N concentration observed in HADG goats may favor microbial protein production. These findings suggest that the rumen microbiota of HADG goats likely had higher fermentation efficiency, supporting the higher ADG of these goats compared with LADG goats.

While most studies have focused on the rumen fluid associated bacteria, it is crucial to acknowledge the distinct microbiota residing in the rumen epithelium (Pei et al., 2010). Malmuthuge et al. (2019) emphasized the inadequacy of studies only based only on rumen contents in capturing the complete rumen microbiome. In our current study, alpha diversity analysis revealed higher bacterial diversity and richness in rumen epithelium compared to rumen fluid. Notably, *Butyrivibrio* and *Prevotellaceae* UCG-001 were predominant in rumen epithelium, contrasting with *Prevotella* and norank_f_F082, which dominated the rumen fluid. However, it is worth noting that diversity indices were reported to be higher in rumen digesta than in mucosal tissue in pre-weaning calves (Malmuthuge et al., 2014), likely due to the differences in growth stage and diet between weaned and pre-weaned ruminants.

The higher bacterial diversity and richness imply a more intricate microbial population and complex host-bacteria interactions within the rumen epithelium. Previous studies have highlighted the involvement of ruminal epithelium bacteria in various crucial processes such as oxygen scavenging, tissue recycling, urea digestion, and anatomic and functional development of rumen (Cheng et al., 1979; Jiao et al., 2015). In our study, we observed that LADG goats had higher abundances of the genera U29-B03 and *Quinella* in the rumen epithelium compared to HADG goats. While U29-B03, a member of the *Rikenellaceae* family, has been reported to positively influence SCFAs production (Du et al., 2021), it is worth noting that Wang et al. (2023) found an unclassified *Rikenellaceae* to be positively correlated with the acetate levels but negatively correlated with growth performance traits in dairy goats. Additionally, Yi et al. (2022) reported a negative correlation between *Quinella* abundance and propionate concentration. When enriched, *Quinella* cells, were found to predominantly ferment glucose into lactate, with minimal production of acetate, propionate, and CO_2_ (Brough et al., 1970). Furthermore, genera U29-B03 and *Quinella* abundances were higher in the rumen fluid of LADG goats compared with HADG goats in the current study. Thus, these findings suggest that the genera U29-B03 and *Quinella* may be the key bacteria influencing the fermentation pattern, which could partly explain the lower propionate concentration observed in LADG goats.

The genus *Desulfovibrio* comprises the predominant sulfate-reducing bacteria in the rumen (Drewnoski et al., 2014; Zhao et al., 2020). Hydrogen sulfide gas (H_2_S) is the principal terminal metabolite produced by *Desulfovibrio* via the dissimilatory sulfate reduction pathway (Rey et al., 2013). In our study, the abundance of *Desulfovibrio* was found to be higher in both the rumen epithelium and fluid in LADG goats compared with HADG goats. Although the relationship between ruminal *Desulfovibrio* and the production performance of ruminants remains unclear, overgrowth of *Desulfovibrio* is known to correlate with several human intestinal and extra-intestinal diseases (Singh et al., 2023). Wu et al. (2021) found that a high sulfur diet increases the ruminal abundance of *Desulfovibrio* and compromises the integrity and barrier function of the rumen epithelium. According to the Spearman correlation analysis, the abundance of *Desulfovibrio* was negatively correlated with ruminal propionate and NH_3_-N concentrations, as well as with the expressions of epithelial tight junction-related genes. Based on these findings, we speculated that *Desulfovibrio* may play a critical role in influencing the growth performance of LADG goats, but further investigations are warranted to clarify the ecological functions of *Desulfovibrio* in the rumen.

In our study, we observed lower abundances of the genus *Alloprevotella* in the rumen fluid and several members of family *Lachnospiraceae* (e.g., *Lachnospiraceae*_ND3007_group and *Lachnospiraceae*_UCG-009) in the rumen epithelium of LADG goats compared with HADG goats. *Alloprevotella,* belonging to the family *Prevotellaceae,* has the capacity to produce moderate amounts of acetate and significant amounts of succinic acid (Downes et al., 2013). Our findings revealed a positive correlation between propionate concentration in the rumen and *Alloprevotella*, consistent with previous research by Fan et al. (2020). Furthermore, Xie et al. (2022) proposed that species within the *Lachnospiraceae* family may play a key role in carbohydrate metabolism and influence feed efficiency in dairy cows. In our study, genera within *Lachnospiraceae* showed positive correlation with ADG, ruminal butyrate concentration, and the expression of genes related to epithelial tight junction. These results suggest that *Alloprevotella* and *Lachnospiraceae* could potentially serve as probiotics, enhancing fermentation efficiency and promoting host health.

We further explored the differences in fungal composition to gain a comprehensive understanding of the rumen microbiota between LADG and HADG goats. We observed a lower abundance of genus *Symmetrospora* and a tendency towards higher abundances of the genera unclassified_f_*Cladosporiaceae* and *Aspergillus* in LADG goats. *Symmetrospora foliicola* has previously been associated with body weight in lambs at 90 days of age (Yin et al., 2023). Moreover, several mycotoxins, such as aflatoxins and ochratoxin A, can be produced by *Aspergillus* fungi and may disrupt ruminal functions (Loh et al., 2020). We also observed a negative correlation between the abundance of *Aspergillus*and and the mRNA expression of *Claudin-4*, suggesting that *Aspergillus* may impair the integrity of the ruminal barrier.

The rumen epithelium serves as a critical site for host-bacteria interactions, acting as the first line of defense against pathogens and facilitating nutrient uptake to support the development and growth of host (Petri et al., 2018). Transcriptome analysis in our study revealed differential expression of 415 genes between LADG and HADG goats, with enrichment in cell junction (*CDH24*, *ITGB2, APOE*, and *ACTN2*), cell adhesion (*SPON2*, *SCARF2*, *VNN1*, and *POSTN*), and others. The tight junction barrier of rumen epithelium plays a pivotal role in preventing microbial invasion and the entry of harmful substances, which is crucial for the growth and health of ruminants (Aschenbach et al., 2019). Our findings indicated lower expression levels of *CDH24, ZO-1,* and *Occludin* in the rumen epithelium of LADG goats. Additionally, *PLVAP* was demonstrated to play a critical role in endothelial barrier function and intestinal homeostasis (Stan et al., 2012). Consistent with our results, studies on monogastric animals have shown lower expression of tight junction proteins in low-birth-weight piglets (Huang et al., 2021; Wang et al., 2023). Furthermore, several bacteria inhabiting the rumen epithelium were significantly associated with the mRNA expression of *ZO-1* and *Occludin* in our study, suggesting that these bacteria may influence the growth performance by regulating the rumen epithelial barrier.

In the current study, the transcriptome analysis showed that cell adhesion related genes, such as *SPON2*, *VNN1*, and *CTSK*, were upregulated in HADG goats. The *SPON2* encodes mind in, which binds to bacteria and their components, acting as an opsonin (He et al., 2004). *VNN1*, which encodes pantetheinase, is involved in coenzyme A metabolism, lipid metabolism, and energy production (Bartucci et al., 2019). Salzano et al. (2023) suggested that the increased *VNN1* expression in rumen epithelium of buffaloes fed a green feed diet was associated with the anti-inflammatory activities. Moreover, the gene *CTSK*, involved in the regulation of developmental processes, has been shown to maintain the normal composition of intestinal microbiota (Sina et al., 2013). However, the functions of these DEGs in ruminants are not well understood, and further research is needed to confirm these findings.

In our study, the mRNA expressions of *NHE-2* and *NHE-3* were found to be higher in HADG goats. The Na^+^/H^+^ exchanger (NHE) proteins regulate the intracellular pH of rumen epithelium by transporting H^+^ to the lumen or extracellular space and taking up Na^+^ into epithelial cells (Schlau et al., 2012). Increased NHE activities are known to lower the local pH near epithelial cells, thereby enhancing the uptake of the undissociated form of SCFAs via simple diffusion (Graham et al., 2007). In addition, the presence of the genus *Oscillospiraceae* NK4A214 group in the rumen epithelium was positively associated with the relative expressions of *MCT-4*, *NHE-2*, *NHE-3*, and *AE2*. Previously classified in the family *Ruminococcaceae*, *Oscillospiraceae* NK4A214 group has been linked to improving growth and lactation performance in ruminants (Tong et al., 2018; Huang et al., 2021; Wang et al., 2023). Consequently, *Oscillospiraceae* NK4A214 group could serve as a potential probiotic to enhance growth or production by mediating SCFAs transport-related genes in the rumen epithelium. However, *Oscillospiraceae* NK4A214 group remains uncultured, and its exact mechanism of action in rumen remains unclear, warranting further investigation.

## Conclusion

**Figure 8:**
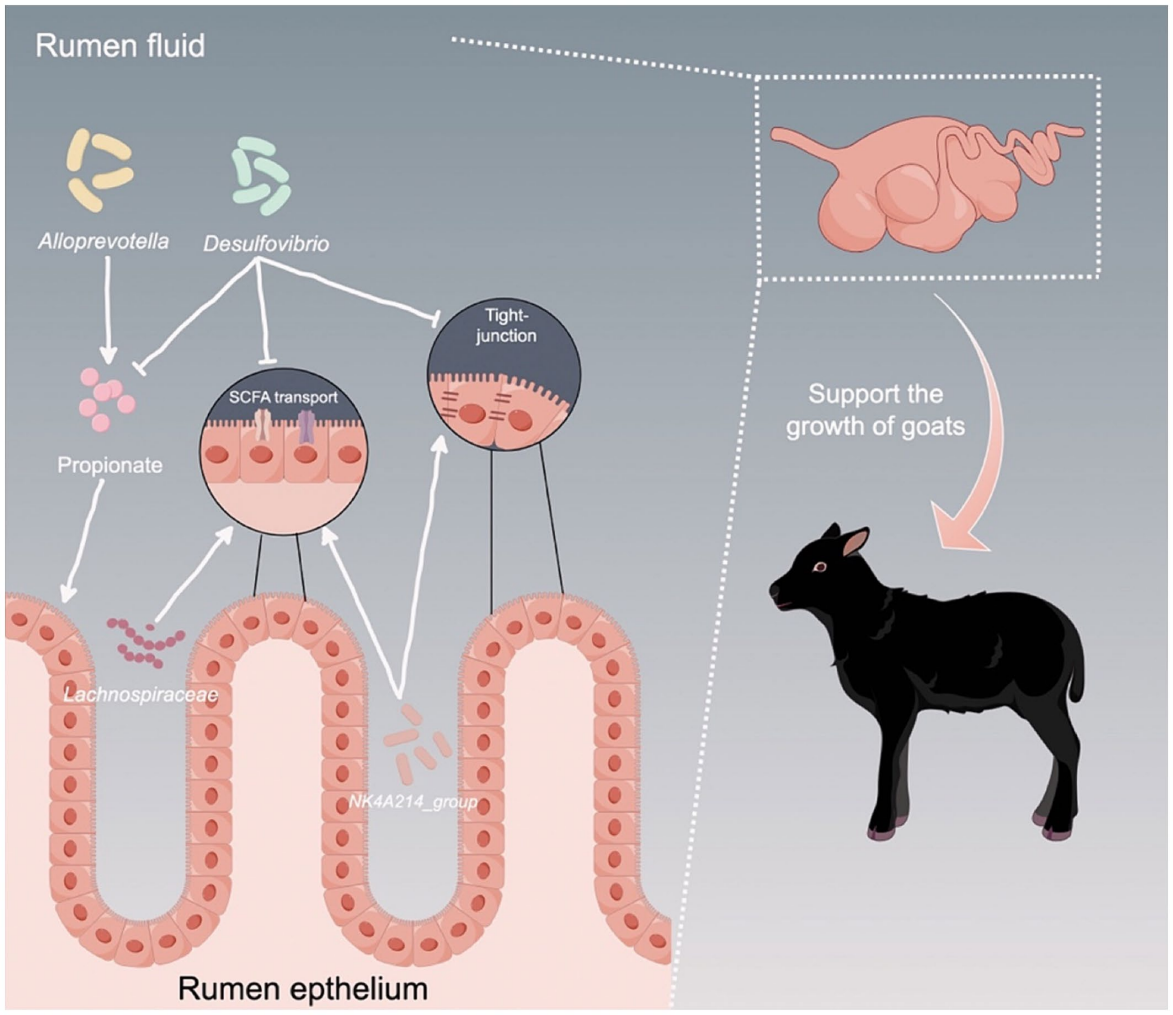
Proposed model of the interactions between ruminal microbiota and the host to support the growth of young ruminants. Arrows indicates upregulation, while T bars indicate downregulation.

In summary, our study reveals that HADG goats exhibit higher ruminal fermentation efficiency, improved rumen epithelial barrier functions, and enhanced SCFAs transport, all of which support the development and growth of goats. These differences can be attributed to the rumen microbiota, particularly to the rumen epithelium bacteria (Figure 8). Thus, our findings offer a deeper understanding of the association between rumen microbiota and growth performance, and identify several potential microbial targets for the development of novel intervention strategies to improve the growth performance of young ruminants.

## Data Availability

The raw sequencing reads of the rumen microbiota, along with the RNA sequencing data, were deposited into the NCBI Sequence Read Archive (SRA) database and NCBI’s Gene Expression Omnibus under the accession numbers PRJNA1099693 and PRJNA1101717, respectively.
