# Supplementary material for: Multi-omics analysis reveals the effects of host-rumen microbiota interactions on growth performance in a goat model
Source: Front Microbiol. 2024 Sep 9;15:1445223. doi: 10.3389/fmicb.2024.1445223 (PMC11417024; doi:10.3389/fmicb.2024.1445223)
Supplement: Supplementary file 1 [file Table_1.docx]

**Multi-omics analysis reveals the effects of host-rumen microbiota interactions on growth performance in a goat model**

**Juncai Chen^a†*^ , Xiaoli Zhang^a†^，Xuan Chang^a^, Bingni Wei^a^, Yan Fang^a^, Shanshan Song^a^, Daxiang Gong^b^, Deli Huang^b^, Yawang Sun^a^, , Xianwen Dong^c^, Yongju Zhao^a^, and Zhongquan Zhao^a*^**

^a^*College of Animal Science and Technology, Chongqing Key Laboratory of Herbivore Science, Southwest University, Chongqing, 400715, China*

^b^*Tengda Animal Husbandry Co., Ltd., Chongqing, 402300, China*

^c^*Chongqing Academy of Animal Science, Chongqing, 402460, China*

***Corresponding author:**

*Email address*: [zhongquanzhao@126.com](mailto:zhongquanzhao@126.com) (Zhongquan Zhao), [juncai.chen@hotmail.com](mailto:juncai.chen@hotmail.com) (Juncai Chen).

**^†^These authors contributed equally to this work**

Table S1. The average daily gain (ADG) of goats

| No. | Group | ADG (g/d) |
| --- | --- | --- |
| 4095 | LADG | 59.74 |
| 4165 | LADG | 61.54 |
| 4132 | LADG | 62.75 |
| 4140 | LADG | 64.04 |
| 4034 | LADG | 70.10 |
| 4120 |  | 70.14 |
| 4171 |  | 72.17 |
| 4164 |  | 73.85 |
| 4151 |  | 75.76 |
| 4107 |  | 76.92 |
| 4121 |  | 78.47 |
| 4180 |  | 80.21 |
| 4159 |  | 80.81 |
| 4166 |  | 81.80 |
| 4170 |  | 83.83 |
| 4073 |  | 84.02 |
| 4056 |  | 85.24 |
| 4138 |  | 86.21 |
| 4123 |  | 86.54 |
| 4195 |  | 87.87 |
| 4187 |  | 88.06 |
| 4150 |  | 88.38 |
| 4168 |  | 88.97 |
| 4023 |  | 89.07 |
| 4145 |  | 90.92 |
| 4131 |  | 91.89 |
| 4105 |  | 91.93 |
| 4089 |  | 92.16 |
| 4201 |  | 92.45 |
| 4126 |  | 92.79 |
| 4158 |  | 92.95 |
| 4194 |  | 93.41 |
| 4094 |  | 93.47 |
| 4088 |  | 93.70 |
| 4172 |  | 93.78 |
| 4127 |  | 93.88 |
| 4186 |  | 94.24 |
| 4167 |  | 94.36 |
| 4144 |  | 94.53 |
| 4169 |  | 95.07 |
| 4177 |  | 95.43 |
| 4125 |  | 95.88 |
| 4098 |  | 96.44 |
| 4161 |  | 96.45 |
| 4092 | HADG | 96.92 |
| 4099 | HADG | 98.49 |
| 4152 | HADG | 98.49 |
| 4103 | HADG | 101.79 |
| 4136 | HADG | 103.45 |
