# Supplementary material for: Multi-omics analysis reveals the effects of host-rumen microbiota interactions on growth performance in a goat model
Source: Front Microbiol. 2024 Sep 9;15:1445223. doi: 10.3389/fmicb.2024.1445223 (PMC11417024; doi:10.3389/fmicb.2024.1445223)
Supplement: Supplementary file 2 [file Table_2.docx]

**Multi-omics analysis reveals the effects of host-rumen microbiota interactions on growth performance in a goat model**

**Juncai Chen^a†*^ , Xiaoli Zhang^a†^，Xuan Chang^a^, Bingni Wei^a^, Yan Fang^a^, Shanshan Song^a^, Daxiang Gong^b^, Deli Huang^b^, Yawang Sun^a^, , Xianwen Dong^c^, Yongju Zhao^a^, and Zhongquan Zhao^a*^**

^a^*College of Animal Science and Technology, Chongqing Key Laboratory of Herbivore Science, Southwest University, Chongqing, 400715, China*

^b^*Tengda Animal Husbandry Co., Ltd., Chongqing, 402300, China*

^c^*Chongqing Academy of Animal Science, Chongqing, 402460, China*

***Corresponding author:**

*Email address*: [zhongquanzhao@126.com](mailto:zhongquanzhao@126.com) (Zhongquan Zhao), [juncai.chen@hotmail.com](mailto:juncai.chen@hotmail.com) (Juncai Chen).

**^†^These authors contributed equally to this work**

Table S2. Primer sequences of selected genes

| Gene | Accession Number | Primer Sequence (5′- 3′) | Product Size (bp) |
| --- | --- | --- | --- |
| ZO-1 | XM_018066118.1 | F:AGCAGACGCAGAAAACCATCA  R: TCTCCACGCCACTGTCAAACT | 225 |
| Claudin-1 | HM_117762.1 | F:CACCCTTGGCATGAAGTGTA  R: AGCCAATGAAGAGAGCCTGA | 216 |
| Claudin-4 | HM_117763.1 | F: AAGGTGTACGACTCGCTGCT  R: GACGTTGTTAGCCGTCCAG | 238 |
| Occludin | BC_133617.1 | F: GTTCGACCAATGCTCTCTCAG  R: CAGCTCCCATTAAGGTTCCA | 200 |
| MCT-1 | XM_013962525.2 | F: ACCAGTTTTAGGTCGTCTCA  R: GGCTTCTCAGCAACATCTACA | 207 |
| MCT-4 | NM_001109980.1 | F:GTTTGGGATAGGCTACAGTGACACA  R: GCAGCCAAAGCGATTCACA | 106 |
| NHE-2 | XM_604493 | F:TTGGAGAGTCCCTGCTGAAC  R: GGCCGTGATGTAGGACAAAT | 225 |
| NHE-3 | NM_174833.2 | F: AGCTACGTGGCCGAGGG  R: AGACAGAGGCCTCCACGGT | 121 |
| AE2 | NM_001205664.1 | F: AGCAGCAACAACCTGGAGT  R: GGTGAAACGGGAGACGAA | 123 |
| IL-1β | XM_013967700.2 | F: GAAGAGCTGCACCCAACA  R: CAGGTCATCATCACGGAAG | 172 |
| IL-6 | NM_001285640 | F: AGATATACCTGGACTTCCT  R: TGTTCTGATACTGCTCTG | 80 |
| NK-κB | XM_005681365 | F: CTGGAAGCACGAATGACAGA  R: GCTGTAAACATGAGCCGTACC | 197 |
| TNF-α | NM_001286442 | F: TGGTTCAGACACTCAGGT  R: CGCTGATGTTGGCTACAA | 75 |
| GAPDH | XM_005680968.3 | F:GCAAGTTCCACGGCACAG  R:GGTTCACGCCCATCACAA | 118 |
| β-actin | NM_001009784.3 | F:GGATGATGATATTGCTGCGCTC  R:TCTCCATGTCGTCCCAGTTGGT | 248 |
